# Engagement of vulnerable communities in HIV prevention research in India: a qualitative investigation

**DOI:** 10.1186/s40900-024-00542-w

**Published:** 2024-01-25

**Authors:** Venkatesan Chakrapani, Vijayalakshmi Loganathan, Paromita Saha, Devi Leena Bose, Nabeela Khan, Tiara Aurora, Jyoti Narayan, Joyeeta Mukherjee, Saif ul Hadi, Chitrangna Dewan

**Affiliations:** 1 Centre for Sexuality and Health Research and Policy (C-SHaRP), 119/2, Mathura Flats, Anna Nagar West Extension, Chennai, 600101 India; 2 IAVI, Unit No. 810, 8th Floor, Emaar Capital Tower – 1, Mehrauli Gurugram Road, Sikandarpur, Sector 26, Gurugram, Haryana 122002 India; 3 Quicksand Design Studio, 7A, Sanskriti Kendra, Anandagram, MG Road, Aya Nagar, New Delhi, 110047 India

**Keywords:** Community engagement, HIV prevention research, Qualitative research, India, Community-academic partnerships

## Abstract

**Background:**

Meaningful community engagement (CE) in HIV prevention research is crucial for successful and ethically robust study implementation. We conducted a qualitative study to understand the current CE practices in HIV prevention research and to identify expressed and implicit reasons behind translational gaps highlighted by communities and researchers.

**Methods:**

For this exploratory qualitative study, we recruited a purposive sample of participants from Indian government-recognised key populations such as men who have sex with men, transgender women, people who inject drugs and female sex workers; general population adults and adolescents/youth; and researchers. We conducted 13 virtual focus groups (n = 86) between July and October 2021. Data were explored from a critical realist perspective and framing analysis (i.e., examining how the participants framed the narratives).

**Results:**

Participants reported that study communities, especially those from key populations, were primarily involved in data collection, but not necessarily with optimal training. Involvement of communities before the start of the study (e.g., obtaining feedback on the study’s purpose/design) or once the study is completed (e.g., sharing of findings) were highlighted as priorities for meaningful engagement. Participants suggested meaningful CE in all stages of the study: (1) before the study—to get inputs in finalising the study design, drafting comprehensible informed consent forms and culturally-appropriate data collection tools, and deciding on appropriate monetary compensation; (2) during the study—adequate training of community field research staff; and (3) after the study—sharing the draft findings to get community inputs, and involving communities in advocacy activities towards converting evidence into action, policy or programs. Timely and transparent communications with communities were explicitly stated as critical for gaining and maintaining trust. Mutual respect, reciprocity (e.g., appropriate monetary compensation) and robust community feedback mechanisms were considered critical for meaningful CE.

**Conclusions:**

The findings highlighted the translational gaps and priority areas for capacity building to strengthen CE in HIV prevention research. It is not only important to engage communities at various stages of research but to understand that trust, dignity, respect, and reciprocity are fundamentally preferred ways of meaningful community engagement.

## Abstract (plain language summary)

Engaging communities in HIV prevention research enhances the rigour and impact of research. We sought to understand the current community engagement practices and to identify how communities preferred to get involved in research. We explored these topics with key and general populations and researchers, by conducting 13 focus group discussions with 86 participants. We found that there was limited involvement of communities before the start of the study and after its completion, although trained community members were involved in data collection. Participants strongly suggested that the community should be involved throughout—before initiation, during the study and after study completion. Participants’ preferred ways of engaging communities reflected that mutual respect, reciprocity and transparent communications are critical for meaningful and successful community engagement.

## Introduction

The success of HIV prevention research and clinical trials among at-risk and vulnerable populations may require strong and equitable community engagement efforts to enhance community understanding, appreciation and participation in biomedical research [1]. Given the sensitive nature of the topic, it is crucial to focus efforts towards building long-term partnerships with and gaining the trust of the study communities. The HIV prevention research field is rapidly evolving to address HIV-related health disparities by using preventive biomedical interventions such as HIV pre-exposure prophylaxis and treatment as prevention strategy, as well as through a wide range of HIV prevention products in the pipeline, including long-acting antiretroviral-based vaginal rings, and broadly neutralising antibodies. Recent efforts directed towards developing a new generation of vaccine candidates and strategies (e.g., germline targeting, viral vectors) have fuelled optimism for improved and effective prevention tools against HIV.

India has the third largest population of people living with HIV along with a ‘concentrated HIV epidemic’ characterised by high prevalence of HIV among key populations such as men who have sex with men (MSM), transgender women (TGW), people who inject drugs (PWID) and female sex workers (FSWs) [2]. Therefore, it is imperative to conduct appropriate community-based studies towards developing scientifically robust and regionally-relevant HIV prevention products as well as informing delivery and uptake of existing and upcoming products as per population needs in India.

The success of any population-based HIV prevention research or trial may also require the acceptance and support of the end-user communities, which can be achieved through meaningful and participatory engagement of communities and stakeholders. To create an enabling environment that ensures ethical conduct and scientific integrity of research, it is imperative to direct efforts towards early and sustained community engagement [3, 4]. With evolving community dynamics and social structures, contemporary stakeholder networks and advancement in technology, it is pertinent to leverage lessons learnt from past research engagement and re-assess the strengths, gaps and challenges. For example, key populations’ increasing access to the Internet via smartphones has presented a way to reach, understand and address their HIV prevention needs as well as engage them in research. In addition, it is important to work out feasible community engagement solutions that incorporate lessons, and address gaps, challenges and strengths and to have a constructive and meaningful community engagement model in place which is suited to the current needs [5].

Several competing definitions of community engagement exist. Given that this study focuses on community engagement in HIV research, we adapted a definition used by the U.S.-based HIV Prevention Trials Network. Community engagement (CE) can be defined as the ways and mechanisms by which the community participates fully at every stage of the research process, expresses opinions and concerns, and influences the research process. There is a dearth of literature in India that reports community engagement experiences or practices in relation to HIV prevention research [6], although studies on community preferences in accessing and using new and emerging HIV prevention products are increasing [7–9]. In this context, this qualitative study was conducted to understand how communities, both key and general populations, have been engaged in HIV prevention research and trials in India; and to identify the gaps and needs from the perspectives of both community representatives and researchers. The study aimed to explore and understand the perspectives and experiences of vulnerable communities and researchers on the engagement of communities in past and ongoing HIV prevention research; and to understand the expectations and aspirations of communities and researchers on how communities need to be involved in research. The study findings are expected to address the critical gap in the literature on community engagement practices in HIV prevention research in the South Asian context and to inform strategies to advance community engagement in HIV prevention research and trials.

## Methods

We used an exploratory descriptive-interpretive qualitative research approach [10] in this study, by focusing on the description and interpretation of the phenomenon of community engagement. Data were collected between July and October 2021 through virtual focus group discussions (FGDs) during the COVID-19 lockdown periods in India. The virtual mode of data collection facilitated recruiting participants across the country, with participants recruited from more than 15 States of India. FGDs were conducted among: (1) key populations which included representatives of MSM, TGW, PWID and FSWs, and their community advocates/leaders; (2) general populations, which included adult men/women, adolescent boys/girls and young men/women (ABYM and AGYW) and pregnant women and nursing mothers (PWNM); and (3) researchers. Purposeful sampling strategy with maximum variation (or diversity) sampling, a subtype of purposive sampling, was used to capture diverse subgroups of key and general populations in terms of age group, gender, education status and identities [11]. Across all the subgroups of communities, the common inclusion criteria were: at least 18 years old, the ability to provide informed consent, and past experience in participating in a research study or knowledge about community engagement practices in HIV prevention research in their communities. The key inclusion criterion for researchers was involvement in HIV-related prevention research over the past five years. Non-governmental organisations (NGOs) and community-based organisations (CBOs) helped in identifying eligible and willing participants through trained peer recruiters. Six FGDs were conducted among key populations, five among general populations and two among researchers.

Each FGD lasted for about 1.5–2 h with assurance of privacy and confidentiality. The participants had the choice of not revealing their real names and had the freedom to turn off their videos during the virtual FGDs. The study protocol was approved by the Institutional Review Board of the Centre for Sexuality and Health Research and Policy (C-SHaRP). All participants, except researchers, received INR 500 (USD 6.6) each as compensation for their time. The informed consent forms to participate in virtual FGDs were sent by e-mails. Participants replied to those mails to provide explicit consent. Further, before the start of the FGDs, the key content of the informed consent form was shared and verbal consent was obtained for participation and audio/video recordings. The participants were provided the option of not displaying any names or using pseudonyms during virtual focus groups and not to switch on their videos, if they did not want to.

### Data collection

Community representatives from vulnerable communities, such as PWID and sexual and gender minorities, were actively involved in recruitment of participants from the study communities. They also co-moderated the FGDs. Three semi-structured topic guides were used: one for community representatives, one for community advocates, and one for researchers. The topic guides explored the understanding and experiences of CE in relation to past or current HIV-related prevention research and trials; good practices, gaps and lessons learned from the CE approaches; and participants’ expectations on how communities need to be engaged in research. The topic guides (questions and potential probes) were pilot-tested with a few representatives from the study populations and were continually revised (‘rolling’ topic guides) by adding new questions and probes as the FGDs progressed. The FGDs were primarily conducted in Hindi or English, although we offered brief translations in other languages (e.g., Tamil) for participants who were not conversant in Hindi or English. In the final focus group with national-level community representatives, we shared the preliminary findings (from the analysis of other FGDs) to get their feedback and recommendations, a form of member checking or respondent validation [12, 13]. Their suggestions were incorporated into the final manuscript.

### Data analysis

FGDs were digitally recorded and translated into English. Data were explored using thematic analysis [14], identification of common patterns or themes across the data, with techniques adapted from two approaches: (1) the framework analysis approach, which uses a pre-determined coding framework based on theory or empirical literature for analysis [15]; and (2) the grounded theory approach, which focuses on developing codes from the data for making inferences [16]. Accordingly, we developed a codebook based on a priori (pre-determined) codes derived from the FGD topic guides and empirical literature on CE as well as emergent codes [17]. Examples of a priori codes include: community advisory board, community inputs, sharing of findings, community representation, and skill-building. Inductive/emergent codes and categories identified from the text were then added to the codebook and used for further coding and categorising. Examples of emergent codes include: engaging gatekeepers, diversity, sustainability, and inclusion of women. The analytic focus was on describing CE practices before, during and after conducting a research study; identifying expectations on CE, and exploring potential reasons for preferences of certain ways of CE. Two coders with adequate experience in qualitative research coded the first two transcripts independently and discussed with each other to arrive at a tentative codebook as described above. During the analysis of the subsequent transcripts, the coders met periodically and resolved any differences in coding and inferences, with guidance from a senior researcher. Constant comparison of data within sources (communities and researchers) and across sources (source triangulation, with FGDs among key and general populations, and researchers) helped in increasing the validity of the inferences [12].

## Findings

### Participants’ profile

A total of 76 community representatives and 10 researchers participated in 13 virtual focus groups. The mean age of the participants from key populations was 34.2 years (SD 8.1), the general population AGYW and ABYM was 22.2 years (SD 1.8) and the general population adults was 29.6 years (SD 5.9) (Table 1). Within the key populations, men who injected drugs (71.4%), MSM (57.1%) and community leaders (55.6%) had relatively higher levels of education (college graduation, i.e., having at least a bachelor’s or master’s degree). Within the general populations, all the adolescents/youth and adults, and 57.1% in the pregnant women group had a college degree. Within the key populations, a majority were employed as NGO/CBO staff (PWID—57.1%, community advocates—44.5%, and TGW—42.8%) and 57.1% of MSM worked in private companies. Among general populations, all adults and a majority of adolescents/youth (60.7%) were working in NGOs, and 71.4% of pregnant women were homemakers. Given that the focus groups were conducted virtually, we could recruit participants from several states in India: Northern India—Uttar Pradesh and Punjab; Eastern and Northeast India—Jharkhand, Bihar, West Bengal, Odisha, Assam, Manipur, Mizoram, Nagaland, Tripura and Sikkim; Western and Central India—Delhi, Madhya Pradesh, Maharashtra and Rajasthan; and Southern India—Andhra Pradesh, Telangana, Karnataka and Tamil Nadu.

**Table 1:** Socio-demographic profile of participants of focus group discussions among key populations and general population adults and adolescents/youth (N = 76)

*Variable*	Key populations(N = 44)						Adolescents/Youth (N = 13)		General population (N = 19)		
	MSM	TGW	PWID—men	PWID—women	FSWs	Community advocates	AGYW	ABYM	PWNM	Adult women	Adult men
Number of participants	7	7	7	7	7	9	7	6	7	6	6
Age (years) Mean (SD)	27.7 (6.1)	28.5 (7.5)	44.8 (5.9)	34.1 (5.0)	31.5 (2.2)	37.5 (7.6)	21.7 (1.3)	22.8 (2.2)	25.2 (3.9)	31.0 (5.6)	33.5 (5.2)

	n (%)	n (%)	n (%)	n (%)	n (%)	n (%)	n (%)	n (%)	n (%)	n (%)	n (%)
*Highest level of education completed*											
5th grade			1 (14.3)		3 (42.8)	2 (22.2)					
8th grade		1 (14.3)		4 (57.1)							
10th grade		2 (28.6)	1 (14.3)	1 (14.3)	2 (28.6)						
12th grade/diploma	3 (42.9)	3 (42.8)		1 (14.3)	1 (14.3)	2 (22.2)			3 (42.9)		
College degree (under/postgraduate)	4 (57.1)	1 (14.3)	5 (71.4)	1 (14.3)	1 (14.3)	5 (55.6)	7 (100)	6 (100)	4 (57.1)	6 (100)	6 (100)
*Occupation*											
Student	2 (28.6)					1 (11.1)	2 (28.6)	3 (50.0)			
Staff in a private agency or business	4 (57.1)	2 (28.6)	3 (42.9)	2 (28.6)		2 (22.2)			2 (28.6)		
NGO/CBO staff	1 (14.3)	3 (42.8)	4 (57.1)	4 (57.1)		4 (44.5)	5 (71.4)	3 (50.0)		6 (100)	6 (100)
Sex worker		1 (14.3)		1 (14.3)	7 (100)						
Begging		1 (14.3)									
Homemaker									5 (71.4)		
Government staff						2 (22.2)					

MSM: Men who have sex with men; TGW: Transgender women; PWID: People who inject drugs; FSWs: Female sex workers; AGYW: Adolescent girls and young women; ABYM: Adolescent boys and young men; PWNM: pregnant women and nursing mothers

Of the 10 researchers who participated in two focus groups, there were representatives from governmental and non-governmental research agencies. Five participants were principal investigators, four were study coordinators and study physicians, and one was a senior research associate.

We describe the experiences and expectations of the participants in relation to CE before, during and after the study implementation, in accordance with study-focused frameworks of CE [18]. The illustrative quotes related to the findings are compiled in Table 2. Each illustrative quote is given an unique id (e.g., Q1, Q2) and the FGD number and the study subgroup (e.g., “FGD11, Community Advocates”) are indicated as well.

**Table 2:** Illustrative quotes for the themes related to community engagement practices or expectations before, during and after a study/trial

CE before a study initiation
*Refining the study’s focus and plan*
Q1 “Planning and hotspot analysis and in which area it [data collection] will happen, there should be a discussion on this [with communities]”. (FG11, Community Advocates) Q2 “Whenever we design any kind of a study, we should definitely consider what community we are targeting and what is our focus area, whom we are going to be speaking to and their flexibility, their availability, their settings where they are comfortable to talk to us or speak or maybe their timings, so these should be considered whenever we design the study”. (FG12, Researchers) Q3 “I think [about] flexibility component [in research design]. That is why I always insist on participatory approaches. We should also be able to get their views on how to design—because that [engagement] will solve almost half of the problems.” (FG6, Researchers) Q4 “Before doing research, among the communities where we will conduct our research, there will be other stakeholders—other civilised people, teachers. So, it is very important to have meetings with them.” (FG10, Adult Women)
*Formalising connections with community agencies*
Q5 “No one can directly come and work with them without an NGO or support [from community members]. Earlier we needed a platform to get associated, like now it is an organisation [a community-based collective of FSWs].” (FG5, FSWs) Q6 “That [Directly reaching key populations] won't be possible now. You have to go through an NGO—only then you can do research”. (FG11, Community Advocates) Q7 “I think this partnership with CBOs is very key because in longitudinal research we are mainly looking for people who would sustain in the research or participate in the research for a long time. So, we see that most of these key populations are also very much connected with the CBOs because they have drop-in-centres—so we could consider conducting our research study within the CBO premises as well.” (FG12, Researchers)
*Train community representatives for better CE*
Q8 “So how should we [community representatives] be and how can we explain the needs of the hijra culture [to researchers] and how should the responses from research institutions be conveyed to hijra people—all of this should be taught to CAB members and they should be given training. They should also be taught as to how they can remain selfless.” (FG2, TGW) Q9 “Not much attention is paid to communities’ capacity building, leadership quality—especially for women”. (FG4, PWID—Women) Q10 “When we talk about CAB, we also need to talk about capacity building of these teams, strengthening them, providing adequate support so that they can make meaningful and community-friendly decisions for their study.” (FG12, Researchers)
*Getting inputs on informed consent forms*
Q11 “Before doing the research, some people will keep 3–4 sessions just to talk to you, just a hangout with [research team]. If they do like that then you get familiar and you feel that you know this person to some extent so the consent that I give [is based on trust].” (FG1, MSM) Q12 “So, usually, we do have an informed consent form on which we take signatures—but they cannot read it. So, we always have to verbally explain the thing. Whatever is there in the consent form, that is in technical language but when we explain it to them, we do it for at the level for lay people.” (FG3, PWID—Men) Q13 “So, you have to keep many things in mind like language—you cannot speak in Hindi over there, you need to take care of Nagpuri or some regional language. When people come, the biggest mistake they make is, they start speaking in English. There are many from rural areas. But in tribal states, you have to take care of language as it’s very important if you want to connect with the youth.” (FG8, ABYM) Q14 “I am talking about verbal communication, direct one-on-one conversation. If there is a community leader who wants to pass on a message to the community, then the community leader should be equipped to communicate with them and explain these scientific terms in simple language.” (FG1, MSM)
*Getting inputs on study tools*
Q15 “I think when we are developing questionnaires, the communities also need to be consulted with, what type of questions will you be including in this research, how do we approach [ask the questions] because there are certain things which we cannot directly approach [ask] the community.” (FG11, Community Advocates) Q16 “A lot of things that we have learnt, we have learnt it from the people—our participants and our community-based investigators. Especially when we were trying to conduct qualitative research—how to ask a question, what language to use, what are the key words, what is the language which is offensive—all of those things we have learnt from our community members.” (FG6, Researchers) Q17 “If you give money [compensation] then it is like [we are] getting respect. I feel that I have done something good and in acknowledgement I have received cash or in kind”. (FG 4, PWID—Women)
*Establishing and engaging the Community Advisory Board (CAB)*
Q18 “What you shared [about CAB] just now, this can happen, if we want that the work is result based. If such a thing is happening in other countries and they are getting good results, then this should be there in India. We should definitely do this and see what difference it will make. We will get to know only after doing it.” (FG9, Adult men) Q19 “If you want to make a committee, then, like you said, we can keep 6, 7 people, if we are doing it in a ward, then we can take that ward’s member, who represents people and another, we can include people from the Anganwadi…Anganwadi sister and we should include an ASHA worker and like, if there is a BDO [Block Development Officer] there, if it is of a block.” (FG10, Adult Women) Q20 “If in research, we want to make any additions or omissions, then we hold a meeting with the entire team representatives or with stakeholders in which we discuss going forward—how we can change our rules or strategy. In CAB meetings, there is a lot of involvement of the community, which is very useful to develop our strategy.” (FG1, MSM) Q21 “So, I was just thinking that if somebody who is a lay person and who knows only Hindi or the regional language then how can they have access to these boards. If you are trying to make it more inclusive then how can we provide them training in regional languages…how can we make them part of the board.” (FG12, Researchers) Q22 “But CAB can handle community dynamics, that is good and that is why they get appointed or they become part of the process. But can they handle the issues of ethics in research correctly?” (FG12, Researchers) Q23 “They (CAB) should answer to each and every question because there are so many questions in our [potential participants] mind about the situation that we are dealing with now, so they should have perfect answers for everything.” (FG13, PWNM)
*Engaging gatekeepers and community influencers*
Q24 “It is important to ask [hijra] Guru [clan leader]. We have been working for so many years in [city] and have our freedom …you have to take the permission of the Guru.” (FG11, Community Advocates) Q25 “So, even if we try to discuss HIV or AIDS, people don’t wish to talk about it due to the shame and stigma associated with it. We faced a lot of problems. We could not collect any data. We went repeatedly and came back empty-handed. After that, we arranged a meeting with a few members. We connected with well-known educated people and consulted a doctor. Through them, we tried to tell people, how important it is to know about AIDS.” (FG7, AGYW) Q26 “We need to meet the members from the community who they listen to. If we directly go to them, they won't listen. If we can make their president [ward member] understand then they will listen since the president will be educated.” (FG7, AGYW) Q27 “They [researchers/counsellors] can…[meet] the *sarpanch*, the main leader of the village. So, first, we will take their [sarpanch’s] help so that they can create awareness as we won't directly force anyone to get HIV test done.” (FG10, Adult Women) Q28 “There is a brothel and if we go for conducting research over there…If we [community leaders] or the other leaders are there only then they give us entry and they [community leaders] explain everything with facts and very nicely, you can participate in a group or even individually. If anybody from outside comes to do research [without involving community leaders] then they will not even gain entry.” (FG11, Community Advocates)
**CE during the study implementation**
*Capacity building for communities on research basics*
Q29 “Of course, they [research agency] did have an orientation of sorts, like orientation of what this research is about, how you go into the field and how to get the answer, how do you direct the communities and all, that brief kind of orientation was, but not in-depth ones, like the actual process of research.” (FG11, Community Advocates)
*Insights from communities on building researchers’ capacity*
Q30 Sensitization training is important and all people who work in this area [should] know the basics…And along with the participation from the community to understand what kind of language they use, how to talk to them, so all that should also be included in the training. (FG3, PWID—Men)
*Insights from communities on building communities’ capacity*
Q31 “You can even train our community to do research. It’ll be a quality work when we do it. We [as study participants] will not hesitate to reveal any details. So, involve the key population members themselves in data collection.” (FG5, FSW) Q32 “Yes, but it [training] wasn’t such a very conventional type of training. It [training] was done through prolonged discussion and sharing of our experiences in the field of drugs in HIV and how we are going to conduct it. So, after following a digest from the main study implementer, I got involved in doing all these things [conducting interviews and focus groups].” (FG3, PWID—Men)
*Involving peers in recruitment and retention*
Q33 “They [women drug users] are very-very scared whether it will be confidential. So, to get [even] a handful of…women drug users, it is usually better…to go through the TI [targeted HIV intervention projects].” (FG11, Community Advocates) Q34 “We make simpler narratives which can be easily communicated to the communities…peer recruiters, on behalf of the research team, would reach to the community in their preferred languages and communicate the research objectives, the eligibility criteria and the procedures and so on.” (FG12, Researchers)
**CE after the study completion**
*Sharing interim findings to get feedback*
Q35 “Feedback is important…if any shortcomings were there [in the study], then try and make it up in the future.” (FG11, Community Advocates) Q36 “Community can also give feedback on how we can benefit the community by this data, so there could be a discussion on that.” (FG3, PWID—Men) Q37 “We conducted a social-economic study of transgender women, so we shared the instruments and methodology with them. After the research, I had a conference hall full of 150 transgender women in front of whom I presented the data and some of them gave me good recommendations that we incorporated [in the final report].” (FG6, Researchers)
*Sharing study findings and outcomes with the study populations*
Q38 “There is research going on in the community and it is completed. I think it should come back to the community—what is the result of the research, what were the findings. Many times, what I see is, people come and go…they do research, they have respondents, they have questions, they have meetings and everything but we never get to see the result.” (FG11, Community Advocates) Q39 “We share the results by calling for a meeting. It is best for them [communities] to know what the outcome of the research is. We make it in a written format and give it to the household. We tell them that this is the result of conducting the research in the community.” (FG7, AGYW) Q40 “Yes, it will be good if sharing it is good. You will win their trust that what happened with me, they worked for one year, two years, then what was the result of that. Many people work and then leave. Their work is done. The persons that they worked with—they [communities] should know what good has happened from the two-year study.” (FG9, Adult men) Q41 “I am an injecting drug user and have been living with HIV for several years now, I have participated in a couple of studies in the past, and articles have been published in [a reputed international journal].” (FG3, PWID—Men)
*Planning for next steps and sustainability*
Q42 “The findings from the research should be able to guide us for the next steps, what we have not achieved. What were the gaps that were there that were not particularly addressed in this implementation—thus, the research document becomes very helpful. Because research comes with evidence and it’s very important for us, for the community to advocate with evidence, with important stakeholders.” (FG11, Community Advocates) Q43 “Participants are expecting that after the completion of research what we are going to do. This is also important, what I realise, in the field.” (FG6, Researchers) Q44 “If there has been a new research study and there is a new medicine and its results are good in other cities then we should be informed of it and vice versa if the results are good in our city then it should be made known to all cities and everywhere so people know that if a particular medicine is not helping them or not curing then it can happen with this other medicine so they can try the new one.” (FG4, PWID—Women) Q45 “In HIV prevention project…these findings will inform our future studies. So, for sustaining this kind of research studies, I feel that the contributions of community members and CBOs are very important. So, I think we should give equal importance to CBOs and community members in HIV prevention research.” (FG12, Researchers)

### CE before initiation of research data collection

Participants across the groups stressed the importance of actively engaging study communities before starting research studies. They reasoned that it is relevant to involve communities in the design stage to understand their perspectives, concerns and priorities regarding the study’s focus and to get their inputs on the tools and consent forms that are appropriate to the local context.

#### Refining the study’s focus and plan

Community leaders indicated that CBOs' inputs should be received to refine the study’s focus and sampling plan (Q1). Researchers emphasised the importance of involving study communities at the design stage itself to plan study implementation (Q2), using participatory approaches (Q3, Q4).

#### Formalising connections with community agencies

Community participants and advocates opined that, in the current context, research will not be possible without the involvement of CBOs and NGOs who work with key populations, indicating the gatekeeping nature of these agencies and the trust study communities may have in them (Q5). A community advocate reported that many research institutions have started formalising collaborations with community agencies by signing a memorandum of understanding or contracts to conduct research with their constituencies or clients (Q6). Researchers too agreed that formalising collaborations with NGOs/CBOs is essential for the smooth conduct of research, especially for long-term projects (Q7).

#### Training community representatives for better CE

Community advocates and community participants recommended training Community Advisory Board (CAB) members on sharing information about the study with their communities and on better engaging in interactions with research institutions (Q8). Women drug users especially stressed the need to build their capacity and leadership skills to represent their communities as research with drug users often excludes or ignores women drug user involvement (Q9). Researchers too noted the need for capacity building of CAB members to improve decision-making in research implementation (Q10).

#### Getting inputs on informed consent forms

Participants elaborated on the need for research teams to make consent forms more understandable to them by clarifying technical terms. For long-term projects, for example, a prospective cohort study for testing a new HIV prevention product, some community representatives suggested having a few ice-breaking sessions with potential research participants to provide adequate information and build trust before getting their consent (Q11). One participant suggested explaining the content of the consent form to potential participants irrespective of the perceived clarity of the language used in the consent form (Q12). The importance of communicating study information in the local/regional languages was also emphasised. For example, adolescent boys and young men from Northeast India talked about the need to use local languages, not English or Hindi (supposedly widely known language across India), in getting consent (Q13). This participant thus conveyed a key point that assumptions should not be made about using English or Hindi in certain regions of India and the need to include non-urban, non-English-speaking eligible study participants. Similarly, participants suggested training community leaders on appropriately communicating about the research with their constituencies (Q14).

#### Getting inputs on study tools

Community representatives across the focus groups emphasised the need to get the inputs of community representatives and CAB to refine study tools such as survey questionnaires or topic guides (e.g., need for certain questions, question wordings) (Q15). Researchers too acknowledged that inputs from study communities as part of qualitative formative research were useful in framing and asking questions in a culturally appropriate manner (Q16). Further, community participants across the groups suggested the need to involve study community leaders/CBOs to arrive at a proper level of monetary compensation. A woman drug user stressed that monetary compensation is for the time and contribution of participants, and should not be seen as charity (Q17).

#### Establishing and engaging Community Advisory Boards (CABs)

There was immense support for the role and establishment of CABs across groups, even from those who had never heard of CAB before (Q18). Given the critical role of the CAB, participants suggested having diverse stakeholders from local/geographical communities (general population) to understand diverse perspectives and to obtain their support (Q19). Community representatives believed that CABs could significantly contribute to the development of new strategies for study implementation. For example, CABs can help in participant recruitment strategies by identifying the hours in which community members will be available for research-related activities (Q20). In addition to CABs, researchers agreed that a trained ‘lay person’ or ‘community member’ should be included in the Institutional Review Board (IRB) (Q21).

Community representatives and researchers presented conflicting views on the role of the CAB. Participants from key populations felt that CABs could serve as the voice of the communities and provide inputs throughout the study. However, the capacity of CAB members to understand research was questioned by a researcher (Q22) who felt instead that CAB members could primarily “handle community dynamics”. Community representatives from PWNM expected CAB members to understand the benefits and risks of the particular research study, necessary to provide inputs and communicate with the communities they represent (Q23).

#### Engaging gatekeepers and community influencers

Getting the support of key gatekeepers, especially those community leaders of key populations, was noted as a facilitator to enrol potential study participants. For example, a community advocate highlighted the importance of obtaining the support of a hijra community leader (Q24). Similarly, adolescent girls and young women noted that stigma related to HIV and sexuality could hinder participation in HIV-related research. Interactions with gatekeepers and communities, however, helped overcome recruitment barriers (Q25).

Participants from the general population emphasised the importance of involving other key stakeholders (such as civil societies and other community opinion leaders like teachers) besides study communities to gain their alignment and support for the research study (Q4). Members of both general and key populations iterated the importance of gaining the support of ‘gatekeepers’. For example, AGYW representatives suggested that local community leaders like ward members need to be first contacted as they are highly influential, and widely respected in the local communities (Q26). Representatives of the adult women group shared a similar perspective (Q27). Community advocates suggested involving community leaders or influencers of key populations (e.g., “Madams”—referring to brothel owners) (Q28).

### CE during the study/trial

Participants emphasised that community agencies and community members can be involved during the data collection phase, with relevant training if required. The importance of involving gatekeepers—mainly community agencies and community leaders—to recruit potential study participants, and the perceived benefits and challenges CAB faced were also discussed.

#### Capacity building for communities on research basics

Community representatives and advocates emphasised that a basic orientation on the research needs to be provided to data collectors from NGOs/CBOs. A basic orientation about the research should be provided to study communities, not just to study participants, so that the community is aware of the research purpose, goals and details of the specific study (Q29).

#### Insights from communities on building researchers’ capacity

Community representatives recommended training researchers to build their socio-cultural competency and understanding of the communities they are working with, including their socio-economic and cultural contexts and lived realities. Specifically, community representatives/advocates called out the need for researchers to be sensitised on issues such as drug use and addiction sex work, same-sex sexuality, and gender identity and for researchers to use community-appropriate language. These trainings and sensitisation were believed to be crucial for building rapport and connections with study communities (Q30).

#### Insights from communities on building their capacity

Community representatives and advocates emphasised that community members need to be employed in data collection as peer researchers, as this can increase the comfort and openness of study participants (Q31). While they appreciated that community members are increasingly involved in collecting qualitative and quantitative data from study communities, a few community advocates raised concerns about the adequacy of training offered to peer researchers involved in data collection (Q32) and suggested that peer researchers be offered comprehensive training, which can be useful not only for that study but for future studies as well. CAB members were expected to convey community perspectives, irrespective of their individual opinions, and provide ongoing critical and helpful comments to the researchers for implementing the study. Participants suggested providing necessary trainings for CAB members in various phases of the study.

#### Involving peers in recruitment and retention

Participants noted that both general and key populations have several concerns related to enrolment in HIV-related studies: the risk of being outed (e.g., as a sex worker or MSM) and judged by others as promiscuous or at high risk for HIV (Q33). Thus, for both key and general populations, participants felt that involving peer recruiters would help in recruitment and retention in longitudinal studies. Besides having peers as recruiters, participants stressed the need for easy-to-understand user-centred communication materials to help recruitment and retention, for which the CAB can provide inputs (Q34).

### CE after the study/trial

Participants offered several suggestions on how communities can be involved once the study is completed: providing feedback on interim findings, sharing the final report/findings, collaborating on the planning of follow-up studies, and offering suggestions on how to increase the positive impact of the study on programs, policies and practice.

#### Sharing interim findings to get feedback

Several community representatives, especially from key populations, emphasised the need for researchers to share interim findings, at the very least with CAB members or key community leaders for their feedback. Feedback was seen as crucial for obtaining community perspectives that may differ from researchers’ perspectives. This additional or contradictory perspective from communities was perceived to potentially enhance the credibility of the findings (Q35). The groups reflected that community feedback can also enhance the understanding of how research findings can be beneficial to communities, what future research studies can be conducted, and what the next steps to improve policies and programmes can be (Q36). A researcher too agreed that feedback from community members helped in the inclusion of relevant recommendations in a research report (Q37).

#### Sharing study findings and outcomes with the study populations

Community representatives noted that even if communities were actively involved before and during the research, the study results or outcomes are often not shared with them (Q38, Q39). Sharing study findings was believed to enhance the community’s trust in the researchers and the research itself, which is especially important for long-term or multi-phase projects (Q40). Further, acknowledging CBOs and key community contributors in reports or papers was considered a good practice. Listing community representatives as co-authors, if they meet the standard co-authorship criteria, in conference presentations and peer-reviewed publications was suggested and examples of the same from past research studies were shared by the community representatives (Q41).

#### Planning for next steps and sustainability

Community advocates felt that the evidence generated from research would strengthen the advocacy initiatives with key stakeholders to improve the situation of vulnerable communities (Q42). A researcher too noted that communities would like to get information from researchers on their next steps after the study completion and sharing of findings (Q43). They especially want the translation of research evidence into action. Community representatives noted that for studies involving new preventive tools or medicines, post-study access of those tools and medicines must be discussed beforehand (Q44). Given that one study may provide ideas for further research, both community representatives and researchers emphasised the need to have long-term collaboration with communities and treat communities as equal partners (Q45).

## Discussion

We found that continuing community engagement efforts towards HIV prevention research in India need to be reinforced with focused efforts towards addressing translational gaps and capacity-building needs. This study revealed that the involvement of communities before the start of the study, for example, to get feedback on the study purpose or plan, or once the study is completed (e.g., result-sharing with communities) needs to be prioritised to enable timely, meaningful and sustained engagement. The findings particularly highlighted the importance of developing and maintaining trust between researchers and communities, respecting and acknowledging the dignity of communities, and practising reciprocity—all of which are crucial for meaningful and effective community engagement [19]. Our study has contributed to addressing the gap in the empirical data on CE processes and practices in HIV prevention research in India [3, 20] and identifying and addressing ethical issues in conducting HIV-related research among key populations [21, 22].

Limited involvement of communities has been reported from other countries as well. For example, one study documented that less than half of the studies supported by the United States National Institutes of Health (NIH) reported any kind of CE activities, and CAB involvement is even less than other CE activities like sharing of findings [23]. In the present study, we found that communities, especially those from key populations, were primarily involved in data collection, often without adequate training. Similar observations were found in studies on CE from other countries, including a US-based study that reported that community members were involved only in “recruiting subjects and collecting data” [24]. In the present study, participants suggested that research institutions should strengthen CAB members’ capacity to contribute to decision-making processes and the peer research staff’s capacity to collect quality data. These suggestions indicate their intentions that communities should contribute meaningfully to the research and possibly reflect balanced reciprocity beliefs [25]. From that perspective, such capacity-building activities will strengthen the trust between researchers and communities and improve the study’s quality and impact. Participants also recommended that the researchers establish CAB with diverse members (e.g., diversity in gender and socio-economic status) to obtain a range of perspectives, reflecting the pluralistic views on the ground [19].

Respect and dignity seem to underlie some of the preferred CE activities reported by the community representatives in the present study. For example, community representatives recommended that appropriate monetary compensation should be decided in consultation with community representatives, as the compensation should not be seen as charity but as an acknowledgement of their experience-based expertise. This interpretation of ours is consistent with the finding reported from a study that female sex workers felt that they were treated with respect based on how the informed consent was taken, the behaviour of the study staff and the assurance of privacy and confidentiality [22].

We found that, as study communities trust community leaders and non-governmental agencies, especially community-led organisations, getting the endorsement of and working in collaboration with such organisations will help in the recruitment and retention of study participants and a way to establish relations with the study communities throughout the study [26, 27]. However, the present study’s participants also noted challenges in getting the support of gatekeepers, especially community leaders of key populations, which in turn pose challenges in study implementation. For example, getting the support of “madams” in the case of female sex workers [28] and “gurus” (masters or senior leaders) in the case of hijras or transgender women [29] may be essential for smooth initiation and implementation of HIV prevention trials or projects [30]. Enrolment of potential participants in a study may depend on the endorsement of community leaders and NGOs/CBOs, highlighting the trust element.

Similarly, the participants’ accounts in the present study indicated that stigma related to HIV and sexuality and restrictions in the movement of women posed barriers to adolescent girls and young women to participate in HIV-related research. Even though we did not explicitly explore this in our study, in addition to gender-related dynamics, class and caste-related power dynamics could also play a role in whether and how local communities are engaged in research [31] and needs to be taken into account.

In the present study, timely and transparent communications with the study communities were explicitly identified as critical for gaining and maintaining trust. Further, the participants emphasised the importance of communicating the study information in local languages, making the consent form more understandable, and explicitly stating the steps to assure confidentiality—in line with findings from other studies from India [3, 22]. The importance of providing comprehensible information may be particularly true among trials in which new HIV prevention products are tested as participants may believe that those products will prevent them from getting HIV (preventive misconception) [32, 33] and might provide a false sense of protection resulting in engagement in more risky practices, a kind of ‘risk compensation’ [33, 34].

### Limitations and strengths

Given the qualitative nature of the study, our aim was not to generalise in a statistical sense but to identify key concepts that are relevant across the settings (i.e., transferability) [12]. Inclusion of both communities and researchers helped us understand perspectives of both stakeholders (source triangulation) [35], and identify points of convergence and differences. A key strength of this study is having a diverse group of community representatives across the country, which was possible through virtual focus groups as the study was conducted during the COVID-19 pandemic periods. Considering the logistics, only those who can speak and understand Hindi or English were predominantly included, and a few community representatives from certain regions (e.g., Tamil Nadu where Tamil language is spoken) had translators to help them communicate their views. NGOs who worked with key and general populations helped in arranging video meetings if participants did not have smartphones or access to the Internet. Thus, we tried to ensure diversity in the study participants, which might have contributed to documenting the ‘polyphonic’ or multiple voices of the communities [36]. Further, we could identify particular challenges faced by specific subgroups (e.g., women drug users), which added to the richness of the data and captured nuances, which are the key advantages of qualitative research. It is possible that even though we conducted 13 focus groups, data saturation (emergence of no new information in additional focus groups) might not have been achieved, given the diversity in the subgroups who participated in these focus groups. Future studies should ensure an adequate number of focus groups can be conducted among each subgroup (at least three in each) so that data saturation can be achieved and comparisons between subgroups can be made [37]. Even though we used source triangulation, conducting in-depth interviews in addition to focus groups might have added more comprehensive information; but given the rapid assessment nature of this study during the COVID-19 pandemic, we restricted to virtual focus groups. Future studies could use multiple qualitative data collection methods (e.g., focus groups and in-depth interviews) or mixed methods, depending on the scope of the study on community engagement.

### Implications

The study results reiterated the usefulness of engaging communities in HIV prevention research and identified potential solutions for improving the engagement of communities in all stages of research. Depending on the nature of tasks and stage of the research, researchers and research institutions need to involve a range of community stakeholders—not only the organisations that work with study communities but also community leaders who are not affiliated with community organisations. In some cases, community leaders from local governments and parents (e.g., in studies among adolescents/legal minors) may need to be involved. Relevant training of community representatives or organisations for effective participation in all stages of research needs to be provided. Especially for peer research staff, who recruit and collect data from study communities, adequate training is needed to ensure data quality and build trust among the study communities. Similarly, appropriate training needs to be provided for the members of CAB, particularly to improve their communication, deliberation and negotiation skills—to contribute to effective decision-making in research processes. Researchers should also ensure adequate diversity in CAB membership—for example, in terms of socio-economic and educational status, gender, language and so on—as relevant. Once the study is completed, the results need to be shared with a range of community stakeholders and the next steps can be planned in collaboration with them.

Researchers need to build their capacities to effectively engage communities and realise that such an engagement is in the best interests of everyone and should not be seen as just ticking off a checklist. Training modules for researchers to improve the skills and competencies related to community engagement, irrespective of or considering the nature or design of HIV prevention-related clinical trials, need to be developed or existing modules need to be tailored. A set of practical guidelines and a self-administered checklist can be developed for researchers to assess how they engage communities in HIV prevention research so that they can improve on areas where community engagement is not optimal. Similarly, a set of indicators of effective community engagement in various stages of research can be developed, which can be used as a monitoring and quality assurance tool by research institutions or funders. Such a tool should not be limited to researcher activities, but also assess how the structures and processes in the research institutions affect effective community engagement. Research funders need to provide adequate funds for community engagement in the research budget. Research institutions should recognise and acknowledge the efforts of the researchers in effectively engaging communities in research, creating a supportive environment at the structural and institutional levels for effective community engagement.

## Conclusion

Our study findings demonstrate that while communities are increasingly involved in HIV prevention research, much more needs to be done. The findings revealed that in any such research, particularly in biomedical prevention trials, it is not only important to engage communities at all stages (planning, implementation and results-sharing) but to take actions to achieve the desired virtues such as trust, dignity, respect, and reciprocity. While the researchers do need to take steps to improve CE in their studies, steps also need to be simultaneously taken at the research institutional level to create a favourable environment for CE and at the funders’ level in allocating sufficient funds for CE, especially in long-term or multi-phase research projects that are typical in HIV prevention research that test new HIV prevention products.

## Data Availability

Relevant de-identified data (in the form of quotes from the participants) are already included in the manuscript. The entire data cannot be publicly shared because of privacy and confidentiality considerations. Data are available from the C-SHaRP Institutional Review Board Coordinator (csharp.india@gmail.com) for researchers who meet the criteria for accessing de-identified, confidential data.

## Abbreviations

ABYM: Adolescent boys and young men
AGYW: Adolescent girls and young women
CAB: Community Advisory Board
CBOs: Community-based organisations
CE: Community engagement
C-SHaRP: Centre for Sexuality and Health Research and Policy
FGD: Focus group discussions
FSWs: Female sex workers
MSM: Men who have sex with men
NIH: National Institute of Health
NGOs: Non-governmental organisations
PWID: People who inject drugs
PWNM: Pregnant women and nursing mothers
TGW: Transgender women
